# Demonstration of Coherent Interference between Acoustic Waves Using a Fiber Ring Resonator

**DOI:** 10.3390/s22114163

**Published:** 2022-05-30

**Authors:** Jee Myung Kim, Junghyun Wee, Kara Peters

**Affiliations:** Department of Mechanical and Aerospace Engineering, North Carolina State University, Raleigh, NC 27695, USA; jkim79@ncsu.edu (J.M.K.); jwee@ncsu.edu (J.W.)

**Keywords:** structural health monitoring, optical fiber, acoustic coupling, coherent interference, fiber ring resonator

## Abstract

Optical fibers were previously demonstrated to propagate and detect acoustic modes that were converted from Lamb waves for structural health-monitoring applications; typically, a fiber Bragg grating sensor in the optical fiber is used to detect acoustic modes. Acoustic modes can transfer from one fiber to another through a simple adhesive bond coupler, preserving the waveform of the acoustic mode. This paper experimentally investigates the coherence of acoustic waves through the adhesive coupler, using a fiber ring resonator (FRR) configuration. This configuration was chosen because the wave coupled to the second fiber interferes with the original wave after it encircles the fiber ring. We performed this experiment using different geometries of optical fibers in the ring, including a standard single-mode optical fiber, a hollow silica capillary tube, and a large-diameter multi-mode fiber. The results demonstrate that the acoustic wave, when transferring through an adhesive coupler, interferes coherently even when the main and ring fibers are of different types. Finally, we demonstrate that the FRR can be applied for sensing applications by measuring the mode attenuations in the ring due to a changing external environment (water-level sensing) and measuring the optical-path length change in the ring (temperature sensing).

## 1. Introduction

**Figure 1:**
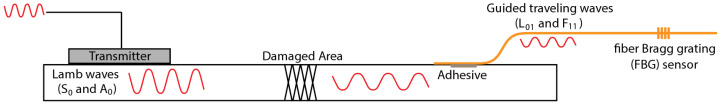
Remote bonding of a fiber Bragg grating sensor structure for guided wave inspection.

Optical fiber sensors are commonly applied to collecting guided waves in structural health-monitoring systems. Recent papers have used fiber Bragg grating (FBG) sensors in a remote-bonding configuration to capture the guided wave [1,2,3]. In this case, the FBG sensor is not in direct contact with the structure, as shown in Figure 1. Instead, the fundamental symmetric (S_0_) and antisymmetric (A_0_) Lamb waves in a structure are converted to propagating fundamental longitudinal (L_01_) and flexural (F_11_) waves in the optical fiber, through an adhesive bond, and are measured with a sensor at a remote location further along the fiber [4,5]. The use of a remote bonding configuration has been shown to increase the sensitivity of the FBG to small-amplitude guided waves [4].

For structural health monitoring applications, it may also be beneficial to capture acoustic waves with different fibers and collect them into a single fiber for processing, or to split the wave from a single fiber into multiple fibers. Early papers demonstrated that these traveling ultrasonic waves can be coupled from one fiber to another through a standard fusion-spliced optical coupler [6,7]. However, fusion-spliced couplers require a precision manufacturing process due to the small wavelength of the light wave (in the order of a single micrometer) and the need to overlap the propagating mode fields between the fibers. These mode fields are confined near their cores; therefore, the fiber diameters need to be reduced in the coupler section. For field applications, adding an optical fiber to an existing sensor network that is bonded to the structure may require removing the existing fiber, then cleaving and splicing it to the coupler leads.

However, in cases where only acoustic coupling between the two fibers is required (and not optical coupling), the authors demonstrated a simpler technique, based on adhesively bonded couplers [8]. The wavelength of the L_01_ mode in a standard optical fiber at ultrasonic frequencies is in the order of a single centimeter; the longitudinal mode is, thus, much more widely distributed in the cross-section of the optical fiber. Therefore, the same precision is not required in the coupler dimensions and the fiber diameter does not need to be reduced. Similarly, Leal et al. [8] demonstrated a fiber-optic acoustic splitter by attaching a segment of optical fiber at two points of another main optical fiber using an adhesive. Using adhesive bonds to couple acoustic waves allows the rapid coupling of the fibers and the extension of new sensors to an already installed sensing system. Since the fibers do not have to be fused, different types and diameters of fibers can also be coupled, meaning that the sensor fiber does not have to be a standard single-mode fiber [9]. Kim et al. [9] demonstrated acoustic-wave transfer via a cyanoacrylate adhesive bond coupler between a polyimide-coated, single-mode optical fiber and different fiber types. These included a single-mode optical fiber, multi-mode optical fibers with different diameters, polyimide-coated silica capillary tubes, and metal fibers. The results published by Kim et al. [9] verified that the waveform of the L_01_ mode is preserved through the adhesive bond coupler and showed that the amplitude of the coupled mode is more complex than that described by the coupled-mode theory. However, the degree of coherence between the original and coupled acoustic waves after the adhesive bond coupler was used was not investigated.

Many optical fiber sensor multiplexing strategies rely on a high degree of signal coherence, including time division and frequency division multiplexing [10,11]. Therefore, to combine the signals from different collection points, it is important that the coherence of the acoustic waves should also be preserved after passing through the coupler. Acoustic waves retain their coherence as they propagate through a structure; therefore, correlating input signals transmitted from different locations with multiplexed signal measurements can be used to extract the contribution of specific sensors individually [12]. The degree of coherence of the ultrasonic waves themselves can also provide input about the integrity of the structure and the presence of scattering due to defects [13]. For this reason, coherence-based multiplexing uses low-coherence input waves to identify the source of each wave [14,15,16]. 

This paper experimentally investigates the coherence of acoustic waves through the adhesive coupler, using a fiber ring resonator (FRR) configuration. This configuration was chosen because the wave coupled to the second fiber interferes with the original wave after it encircles the fiber ring [17,18,19,20]. In addition, the FRR configuration allows us to estimate the coupling coefficient and coupling loss of the adhesively bonded coupler. We performed this experiment using several different geometries of optical fibers in the ring, including a standard single-mode optical fiber, a hollow silica capillary tube, and a large-diameter multi-mode fiber. Finally, we demonstrate that the FRR can be applied for sensing applications by measuring the mode attenuation in the ring due to a changing external environment (water-level sensing) and measuring an optical path-length change in the ring (temperature sensing). 

## 2. Materials and Methods

**Figure 2:**
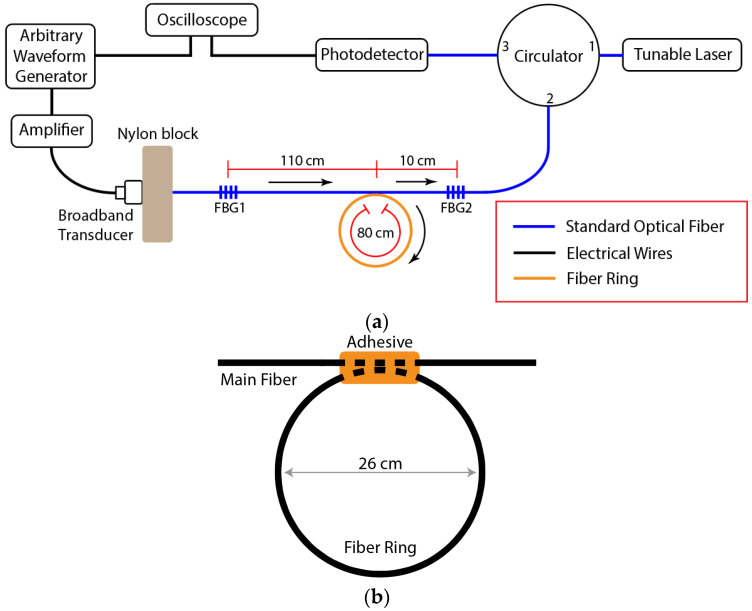
(**a**) Setup of the fiber-ring resonator experiment, with the acoustic wave paths shown as arrows. (**b**) Sketch of the fiber ring, attached to the main fiber using adhesive.

The experimental setup for testing acoustic FRR is shown in Figure 2a. A standard 125-μm diameter single-mode silica fiber with a polyimide coating was used as the main fiber (Micron Optics os1100, Atlanta, GA, USA). The main fiber had FBG sensors on each side of the fiber ring to measure the amplitude of the acoustic modes. FBGs 1 and 2 were 10 mm long and had Bragg wavelengths of 1584 nm and 1616 nm, respectively. One end of the main fiber was connected to the tunable laser (NetTest 3642 HE CL, Peabody, MA, USA) and photodetector (New Focus 1544, Milpitas, CA, USA) via a circulator. The output response from each FBG to the L_01_ acoustic mode was measured by tuning the tunable laser output to the rising edge of the FBG spectrum and then measuring the change in amplitude of the reflected signal. The edge filter was tuned, then the rising-edge slope was calibrated for each FBG, prior to every experiment.

The FRR ring was fabricated by cleaving two ends of a fiber and splicing them into a ring shape. Then, the FRR ring was adhesively bonded to the main fiber, as shown in Figure 2b. To ensure consistency of the adhesive quality, the main fiber and FRR were positioned and taped down to a metal surface, such that a few millimeters in the length of each fiber would align and be in contact with one other, then a single droplet of cyanoacrylate adhesive (Loctite Ultragel Control) was applied to the region of contact. 

The acoustic wave coherence was investigated in terms of the fiber ring resonator for three different ring fibers: a standard 125 μm-diameter single-mode fiber with a polyimide coating, a 220 μm-diameter multimode silica fiber with a polyimide coating, and a 126 μm-diameter silica capillary tube with a 75 μm-diameter hole and polyimide coating. The standard single-mode fiber was selected to examine the wave transfer between identical fibers, while the other two fibers were selected to examine the wave transfer from the single-mode fiber to a fiber with different cross-sectional geometry. 

Sinusoidal, ultrasonic L_01_ modes were launched into the main optical fiber, using a broadband transducer (Olympus C407, Waltham, MA, USA) with varying excitation frequencies from 700 kHz to 730 kHz. Different numbers of cycles were excited (from 1 to 200), depending on the experiment. The burst period for the waveform generator was set to 10 ms, to avoid any possible interference with the reflection of the preceding excitation. The transducer was attached to a nylon block, then one end of the main fiber was glued into a hole that was punctured into the opposite surface of the nylon block. The input excitation signals from the arbitrary waveform generator (AWG) were time-synchronized with the measurement acquisition by sending a TTL trigger signal to the oscilloscope (Agilent Technologies DSO5032A, Santa Clara, CA, USA).

## 3. Results

### 3.1. Investigating Acoustic Wave Coherence Using the FRR

**Figure 3:**
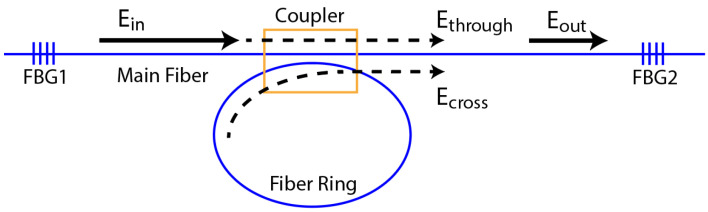
Schematic of the wave flow in FRR for a standard single-mode fiber ring.

The behavior of the acoustic FRR was analyzed using the Sagnac-effect-based resonant fiber optic gyro model derived by Ying et al. [21] for an optical system. This formulation was chosen for our analysis of the passing of acoustic waves through the adhesive bond because it includes a coupling loss coefficient for the coupler. For fused optical couplers, this loss might be negligible; for the adhesively bonded coupler, it is expected to be significant. Figure 3 shows the acoustic wave pathways through the FRR. 

The input wave to the system, $E_{in}$, is measured with FBG 1. In the model of Ying et al. [21], the input wave amplitude is defined as a function of time:

(1) $$E_{in}\left(t\right)=E_{0}e^{\left[2\pi i\left(f_{0}+\frac{kt}{2}\right)t\right]}e^{\left(i\psi_{0}\right)}$$

where $E_{0}$ is the amplitude of the wave, $f_{0}$ is the input frequency, *k* is the frequency sweep rate of the wave and $\psi_{0}$ is the initial phase of the wave. The wave then reaches the FRR and a portion passes directly through the main optical fiber without going through the FRR, $E_{through}$:

(2) $$E_{through}\left(t\right)=E_{0}e^{\left[2\pi i\left(f_{0\;}+\frac{kt}{2}\right)t\right]}e^{\left(i\psi_{0}\right)}\times {\left(1-k_{c}\right)}^{\frac{1}{2}}{\left(1-a_{c}\right)}^{\frac{1}{2}}$$

where $k_{c}$ is the coupling coefficient of the coupler and $a_{c}$ is the coupling loss coefficient. These two coefficients are defined as the fraction of the intensity of the wave, either coupled or dissipated, which is why Equation (2) includes the square root of these terms. The portion of the wave entering the fiber ring is expressed as:

(3) $$E_{ring}\left(t\right)=E_{0}e^{\left[2\pi i\left(f_{0}+\frac{kt}{2}\right)t\right]}e^{\left(i\psi_{0}\right)}\times {\left(k_{c}\right)}^{\frac{1}{2}}{\left(1-a_{c}\right)}^{\frac{1}{2}}$$

and the remaining portion is dissipated through the coupler loss. The wave $E_{ring}$ circles the FRR, and a portion is recoupled into the main optical fiber. 

The portion remaining in the FRR circles the FRR through multiple passes, each time coupling a portion of the wave into the main fiber, until the amplitude of the wave is negligible. Each time the wave propagates through the ring it is attenuated, due to the propagation loss per unit length in the fiber ring, $a_{L}$, and is phase-shifted due to the optical path length of the ring. The value for $a_{L}$ in a standard, polyimide-coated single-mode optical fiber for the L_01_ mode at 300 kHz was measured by Wee et al. [4] to be $0.19\;m^{-1}$. The sum of the waves exiting the FRR with each pass is labeled as $E_{cross}$. After the *N*th pass through the fiber ring, the resulting $E_{cross}$ is expressed as:

(4) $$E_{\mathrm{cross}}\left(t\right)=E_{0}k_{c}\left(1-a_{c}\right){\left(1-a_{L}\right)}^{\frac{1}{2}}e^{\left[2\pi i\left(f_{0}+\frac{kt}{2}\right)t\right]}e^{\left(i\psi_{0}\right)}e^{\left(i\pi \right)}\times \sum_{n=1}^{N}{\left[{\left(1-k_{c}\right)}^{\frac{1}{2}}{\left(1-a_{c}\right)}^{\frac{1}{2}}{\left(1-a_{L}\right)}^{\frac{1}{2}}\right]}^{n-1}\times \;e^{\left[-2\pi i\left(f_{0}n\tau +kn\tau t-\frac{kn^{2}\tau^{2}}{2}\right)\right]}.$$

The transit time is calculated as $\tau =c_{0}L$, where *c*_0_ is the velocity of the acoustic mode in the ring fiber and *L* is the length of the ring. The velocity of the acoustic mode in each fiber type was previously measured experimentally using laser Doppler vibrometry by the current authors [9]. The output wave amplitude after the FRR is the total of these two contributions, expressed as:

(5) $$E_{out}\left(t\right)=E_{through}\left(t\right)+E_{cross}\left(t\right).$$

**Figure 4:**
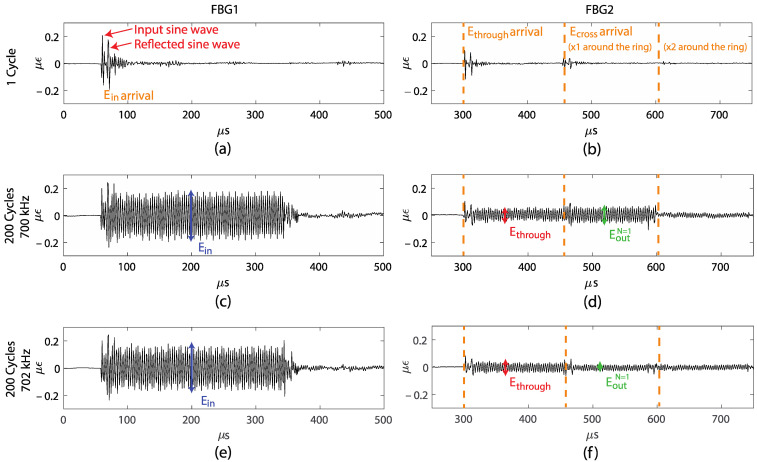
(**a**) FBG1 measurement for 1 cycle per burst, (**b**) FBG2 measurement for 1 cycle per burst, (**c**) FBG1 measurement for 200 cycles per burst at 700 kHz, (**d**) FBG2 measurement for 200 cycles per burst at 700 kHz, (**e**) FBG1 measurement for 200 cycles per burst at 702 kHz, and (**f**) FBG2 measurement for 200 cycles per burst at 702 kHz.

Initial experiments were performed with standard single-mode fiber as the ring and a 700 kHz and 702 kHz excitation signal. Figure 4a–f show the recorded signals at FBG1 and FBG2 for these experiments. Figure 4a shows $E_{in},$ measured by FBG1 for 1 cycle of the sine-wave input. At around 60 μs, we observed the input sine wave, and after approximately 10 μs we observed a second sine wave, which is a reflection that is possibly caused by the wave traversing through the nylon block. Figure 4b shows the FBG2 reading for the same excitation, with the waves $E_{through}$ and $E_{cross}$ after the first pass through the ring, and $E_{cross}$ after the second pass through the ring. The theoretical arrival time for each signal is marked in Figure 4b, confirming the identification of each wave. As the velocities of the acoustic modes are much slower than the optical light waves in optical fibers, the separation time is visible in the measurements and is much larger than the burst duration. Therefore, no interference occurs between $E_{through}$ and the multiple $E_{cross}$. In addition, we can see the actual waveform in the signals. However, from this experiment, we observed that the amplitude of $E_{cross}$ after the second pass through the ring is small compared to the other wave packets; therefore, we only considered $E_{cross}$ after the first pass in the subsequent experiments. This rapid decay is due to the loss in the coupler and the propagating loss in the fiber ring, which are both significant for these experiments.

Figure 4c shows the input wave to the FRR, as measured by FBG1, when the number of cycles per burst is increased to 200, thus increasing the duration of the wave packet to approximately 300 μs. The double sine wave input seen in Figure 4a is repeated as the number of cycles of the sine wave is increased, as seen in Figure 4c–f. Although not as ideal as a sinusoidal wave, the periodic form allows the wave packets to interfere. The amplitude of the input signal $E_{in}$ is shown in Figure 4c. Since the duration of $E_{in}$ was increased, the wave packet of $E_{through}$ partially overlapped and interfered with the wave packet of $E_{cross}$. In Figure 4d, the amplitude of $E_{through}$ and the amplitude of the signal when $E_{out}$ represents the interference outcome of $E_{through}$ and $E_{cross}$ are shown. To demonstrate that interference is actually occurring, the frequency of the input wave was changed slightly. Figure 4e,f show the same FBG1 and FBG2 measurements but, at 702 kHz, there is an input excitation. In Figure 4d we can observe constructive interference as $E_{through}$ overlaps with $E_{cross}$ and, in Figure 4f, we can observe the destructive interference. 

We next used the theoretical model to estimate the coupling coefficient and coupling loss coefficient for the coupler. From Figure 4b, we can observe that the amplitude of the wave decreases significantly when N is greater than 2; thus, we only theoretically model the wave interference of the acoustic FRR for N=1. Since each measurement is taken at a fixed frequency, the sweep rate, *k*, is zero and the input wave equation becomes:

(6) $$E_{in}\left(t\right)=E_{0}e^{\left[2\pi if_{0}t\right]}e^{\left(i\psi_{0}\right)}.$$

Setting N=1, we find the power ratio of the output and input wave by:

(7) $${\left|\frac{E_{out}^{N=1}}{E_{in}}\right|}^{2}={\left|{\left(1-k_{c}\right)}^{1/2}{\left(1-a_{c}\right)}^{1/2}-k_{c}\left(1-a_{c}\right){\left(1-a_{L}\right)}^{1/2}e^{-2\pi if_{0}L/c_{0}}\right|}^{2}$$

where the only unknown parameters are *k_c_* and *a_c_*. Therefore, we can use the measurements at two different frequencies to fit the values of *k_c_* and *a_c_.* Since the full waveform can be measured, in contrast to optical measurements, we do not need to average the amplitude ratio over time; instead, we can determine the amplitude directly from the waveform.

**Figure 5:**
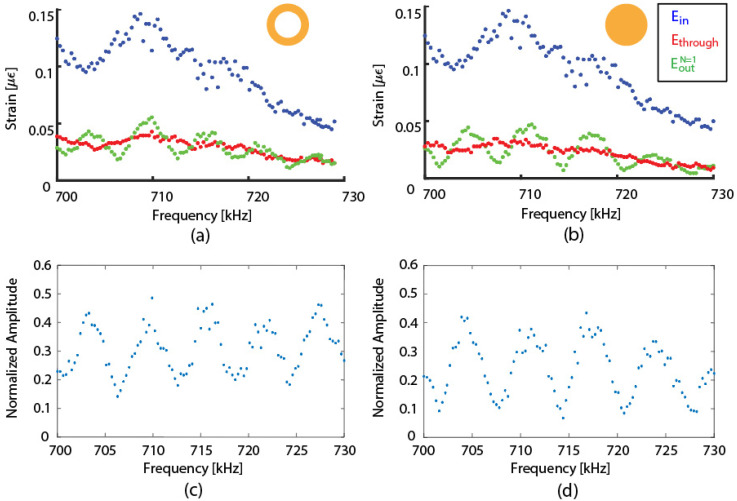
Wave interference pattern from 700 kHz to 730 kHz for: (**a**) a 75-μm hollow-core fiber ring and (**b**) a standard single-mode fiber ring, and normalized wave interference pattern from 700 kHz to 730 kHz for (**c**) a 75-μm hollow-core fiber ring and (**d**) a standard single-mode fiber ring.

Figure 5a,b plot the peak-to-peak amplitude measurements of $E_{in}$, $E_{through}$, and $E_{out}^{N=1}$ as a function of the input frequency $f_{0}$. Figure 5a shows the result for the 75 μm hollow-core fiber, while Figure 5b shows the result for the standard 125-μm single-mode solid fiber. Between the two experiments, the ring was removed, and the standard single-mode fiber ring was attached to the main optical fiber. Therefore, the coupling to the broadband transducer and $E_{in}$ remained the same. The amplitude of $E_{in}$ varied with frequency because the output amplitude from the broadband transducer varied; the wave coupling can vary with the frequency at the transducer to the nylon block interface and at the nylon block to the main fiber interface. Based on these data sets, we examined the wave interference pattern, as shown in Figure 5c,d in which the $E_{out}^{N=1}$ interference pattern is normalized with $E_{in}$.

The results in Figure 5c,d resemble the frequency response of an optical interferometer. In fact, the free spectral range can be calculated using the exponential term in Equation (7), in which the frequency values that satisfy the condition $f_{0}L/c_{0}=n$ for all positive n integers mark the locations of the peaks. Using the length of the FRR and the velocity of the $L_{01}$ mode, the free spectral range of the 75-μm hollow-core fiber ring is 5.1 kHz, while the free spectral range of the standard single-mode fiber ring is 6.5 kHz, which is consistent with the experimentally measured data in Figure 5c,d. Therefore, the wave before and after the coupler interfered coherently.

We next fitted the data from the measurements at the two frequencies into Equation (7), to find the $k_{c}$ and $a_{c}$ for each fiber type. The results are shown in Table 1. The coupling coefficient, $k_{c}$, varied from approximately 0.6 to 0.8 and increased with the fiber silica cross-sectional area of the ring fiber. This behavior was consistent with the previous experiments by Kim et al. [9]. The coupling loss, $a_{c}$, was significant for all cases, as expected. In particular, the value for the 220 μm solid fiber ring was much larger than in the other cases, which may be due to the significant fiber size mismatch between the two fibers. In addition, the ring fiber was bent into a circular configuration prior to bonding; therefore, the large-diameter fiber probably put more stress on the adhesive bond. These results show that the property of the acoustic FRR system can be tuned, based on selecting different fiber ring geometries. 

### 3.2. Demonstration of Acoustic FRR as Sensors

Based on the experiments of the previous section, an FRR can easily be fabricated and attached to any existing sensing fiber using the adhesive coupler, to function as a separate sensor. To show the function of an FRR as a sensor, two demonstrations were performed. One demonstration measures the mode attenuation in a ring in response to a changing external environment, due to a changing water level, and one measures an optical path-length change in the ring due to temperature. Note that these demonstrations were only conducted to show possible applications, and that they were not optimized for practice.

The first demonstration utilized the acoustic FRR as a water-depth sensor, based on the amplitude of the signal from the ring fiber. The acoustic wave in the waveguide attenuated faster when the external medium was water rather than air; so, depending on the amount of fiber ring being submerged in water, the interference pattern will change. For this demonstration, a standard single-mode fiber ring was used. The setup is identical to Figure 2; however, the fiber ring was put inside a water tank and the measurements were taken as the water level was incrementally increased. The input wave was set at 703 kHz frequency, so that the interference pattern would be constructive.

**Figure 6:**
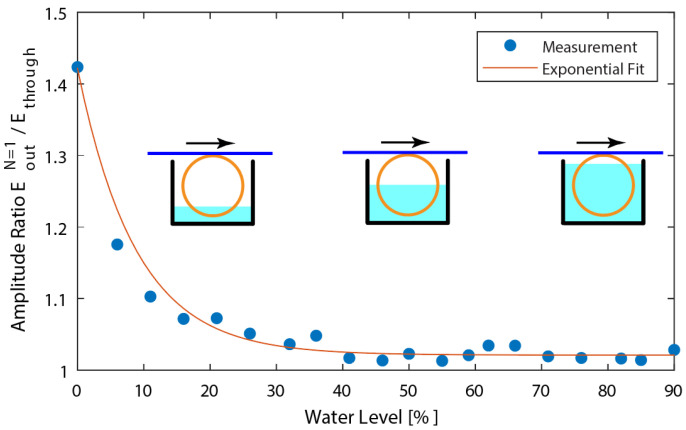
Normalized wave interference pattern for a standard single-mode fiber ring at water levels of 0% to 90%.

Whereas the original coefficient of attenuation around the fiber ring was $a_{L}$, the attenuation coefficient is now expressed as:

(8) $$a_{L}+\frac{L_{w}}{L}\left(a_{w}-a_{L}\right)$$

where *L_w_* is the length of the fiber that is submerged in water. Figure 6 shows the measured $E_{out}^{N=1}$ interference pattern for water levels from 0% to 90%, along with an exponential curve fitted to the data. The output is normalized to $E_{through}$, so that the value of 1 corresponds to there being no energy remaining after the wave travels around the fiber ring. As the water level increased, the amplitude of $E_{cross}$ reduced rapidly; thus, the amplitude ratio decreased, as expected. The sensor output was saturated when approximately 40% of the ring was submerged, confirming that the attenuation in water is considerably higher than that in air.

The second demonstration utilized the acoustic FRR as a temperature sensor, using the coherent interference of the acoustic wave. The standard single-mode fiber ring from Figure 2 was put inside an insulated container and the output amplitude was measured in a frequency range from 700 kHz to 715 kHz. Measurements were performed at three different temperatures: room temperature (21.0 °C), hot temperature (40.3 °C), and cold temperature (11.6 °C). First, the measurements were taken at room temperature, then the temperature was raised to the appropriate hot temperature by placing heat packs inside the container. The FRR was left in the container for 1 h before taking measurements so that it could adjust to the hot temperature. After cooling the FRR back down, the measurements at room temperature were taken again; then, the temperature was lowered to the cold temperature by placing ice packs inside the container. The FRR was left in the container for 1 h, again, before taking measurements. The temperature inside the container was independently measured with a thermometer.

**Figure 7:**
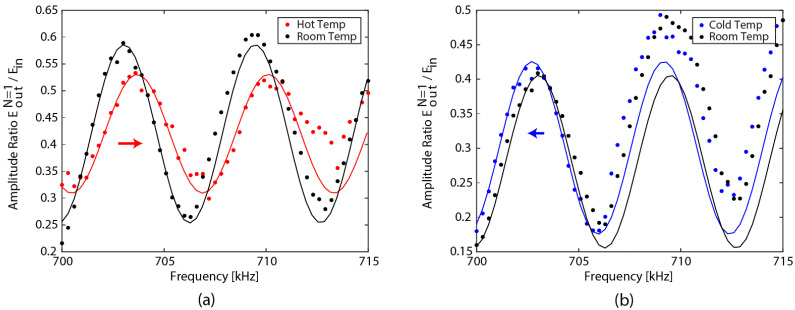
Wave interference pattern and fitted curves from 700 kHz to 715 kHz: (**a**) at room temperature and the hot temperature, and (**b**) at room temperature and the cold temperature.

Figure 7 shows the frequency response measurements and their fitted curves. Figure 7a shows the frequency response for room temperature and the hot temperature, while Figure 7b shows the frequency response for room temperature and the cold temperature. In Figure 7a, the measurement of the hot temperature is noisier than the other three measurements, due to the physical interference of the hot pack inside the container; the FBG was hanging in mid-air during measurements. A fast Fourier transform was performed on the four wave interference patterns to find the free spectral range, which was identical at 6.45 kHz. Using this information, sinusoidal curves were fitted to the frequency response, from which the peak frequency shift was found for each temperature change. The amplitude of the measurements drifted during the experiments, potentially due to changes in the support condition and the resulting contact with the coupler. However, the input frequencies for the peak locations were still the same between the fitted curve and the measured curve. For the temperature change of +19.3 °C (from room temperature to the hot temperature) the peak frequency increased by 628 Hz and, for a temperature change of −9.4 °C (from room temperature to the cold temperature), the peak frequency decreased by 314 Hz. This result shows that the shift in the wave interference pattern is directly related to the change in the environmental temperature, and the calculated sensitivity value is 33.0 Hz/°C.

## 4. Discussion

The experimental results in this paper demonstrate that the acoustic waves transferring through an adhesive coupler interfere coherently. The degree of coherence was not estimated because the coupling loss was significant; however, the output of the FRR can be used for sensing applications that require interference in the input and output acoustic waves, as demonstrated with the temperature measurement experiment. Although the losses in the acoustic FRR, based on the adhesive bond coupler, are high, a unique feature is that the ring fiber used for measurement in the environment does not have to be a standard optical fiber. The ring fiber could be a different material that reacts differently to the environment. Another possibility would be to fill the hollow-core fibers with liquids or gases, which allows even more opportunities for sensing applications. Although different fiber diameters and geometries were used as fiber rings in this paper, there is still a significant amount of work that should be conducted to further analyze and understand the geometrical properties that govern $k_{c}$ and $a_{c}$, and how these can be varied to tune the output of the coupler and the FRR.

## Figures and Tables

**Table 1:** Empirical $k_{c}$ and $a_{c}$ for a 75-μm hollow-core fiber ring, a standard single-mode fiber ring, and a 220-μm solid fiber ring.

75 μm Hollow Core Fiber Ring (8051 μm^2^ Silica)		Standard Single-Mode Fiber Ring (12,271 μm^2^ Silica)		220 μm Solid Fiber Ring (38,013 μm^2^ Silica)	
$k_{c}$	$a_{c}$	$k_{c}$	$a_{c}$	$k_{c}$	$a_{c}$
0.5955	0.7430	0.7181	0.7939	0.8219	0.9494

## Data Availability

Not applicable.
